# Real-world risk of brain metastases in stage III non-small cell lung cancer in the era of PET and MRI staging

**DOI:** 10.3389/fonc.2023.1139940

**Published:** 2023-03-24

**Authors:** Saud Alhusaini, Tyler A. Lanman, Ryan B. Ko, Kate E. Therkelsen, Rie Von Eyben, Maximilian Diehn, Scott G. Soltys, Erqi L. Pollom, Alexander Chin, Lucas Vitzthum, Heather A. Wakelee, Sukhmani K. Padda, Kavitha Ramchandran, Billy W. Loo, Joel W. Neal, Seema Nagpal

**Affiliations:** ^1^ Division of Neuro-oncology, Department of Neurology and Neurological Sciences, Stanford Cancer Institute, Stanford, CA, United States; ^2^ Department of Radiation Oncology, Stanford Cancer Institute, Stanford, CA, United States; ^3^ Division of Oncology, Department of Medicine, Stanford University, Stanford, CA, United States

**Keywords:** non-small cell lung cancer, brain metastases, incidence, surveillance, MRI brain

## Abstract

**Objective:**

The 2-year incidence of brain metastases (BrMs) in stage III non-small lung cell cancer (NSCLC) has been estimated to be around 30%. However, recent clinical trials have demonstrated considerably lower BrMs rates in this patient population. In this study, we aimed to review the real-world incidence, surveillance, and treatment patterns of BrMs in stage III NSCLC.

**Materials and methods:**

Using a retrospective single-center study design, we identified patients with stage III NSCLC who received radiation with curative intent over a 10-year period. Outcome variables included BrMs incidence, overall survival (OS), and survival from date of BrMs. Additionally, we assessed patterns of BrMs surveillance in stage III NSCLC and treatment.

**Results:**

We identified a total of 279 stage III NSCLC patients, of which 160 with adequate records were included in the final analyses [adenocarcinoma (n = 96), squamous cell carcinoma (n = 53), other histology subtype (n = 11)]. The median OS for the entire cohort was 41 months (95% CI, 28-53), while the median time from BrMs to death was 19 months (95% CI, 9-21). Twenty-three patients (14.4%) received planned surveillance brain MRIs at 6, 12, and 24 months after completion of treatment. The remaining 137 patients (85.6%) received brain MRIs at systemic recurrence (restaging) or when neurologically symptomatic. A total of 37 patients (23%) developed BrMs, with a 2-year cumulative BrMs incidence of 17% (95% CI, 11-23). A higher incidence of BrMs was identified in patients with adenocarcinoma relative to those with squamous cell carcinoma (*p* < 0.01). Similarly, a higher 2-year BrMs incidence was observed in patients who received planned surveillance brain MRI relative to those who did not, although statistical significance was not reached. Stereotactic radiosurgery (SRS) treated 29 of BrMs patients (78.4%) and was preferred over WBRT, which treated only 3 patients (8.1%).

**Conclusions:**

At our center, BrMs incidence in stage III NSCLC patients was lower than historically reported but notably higher than the incidence described in recent clinical trials. Routine BrMs surveillance potentially allows earlier detection of asymptomatic BrMs. However, asymptomatic BrMs were mostly detected on restaging MRI at the time of recurrence.

## Introduction

Lung cancer is the leading cause of cancer death worldwide, accounting for 23% and 24% of all deaths by cancer in females and males respectiely (1). Non-small cell lung cancer (NSCLC) accounts for the majority of cases, constituting approximately 76% of all lung cancers (2). Patients with stage III NSCLC have no brain metastases (BrMs) at the time of diagnosis but are at high risk for developing BrMs during their disease course. Despite recent treatment advances, BrMs are a common manifestation of recurrence in stage III NSCLC and often associated with a decreased quality of life and poor prognosis (3–5). The incidence of BrMs in stage III NSCLC has historically been estimated to be around 30% at 2-years (4, 6). However, recent clinical trials have demonstrated considerably lower BrMs rates in enrolled stage III NSCLC patients. Specifically, the PACIFIC trial (PD-L1 inhibitor, Durvalumab vs. placebo for stage III NSCLC) reported a BrMs rate of 11.8% in the placebo arm and 6.3% in the treatment arm at a median follow-up period of 25.2 months (7). It is worth noting however that in this multi-center international clinical trial, fewer brain magnetic resonance imaging (MRI) may have possibly been performed relative to standard practice in US-based health care systems.

With recent advances in diagnostic imaging and rapid accessibility to brain MRI, the true incidence of BrMs in stage III NSCLC in a real-world (outside the strictly controlled clinical trials) setting remains unclear. Given that up to one third of stage III NSCLC patients often develop BrMs over their disease course, our local practice has evolved to performing surveillance brain MRI at 6, 12, and 24 months after completion of initial treatment. Nonetheless, the support for routine asymptomatic BrMs surveillance in this patient population remains to be demonstrated.

In this study, we aimed to determine the real-world incidence of BrMs in stage III NSCLC and analyze the surveillance and treatment patterns of BrMs in this patient population.

## Materials and methods

### Patients and data collection

**Figure 1:**
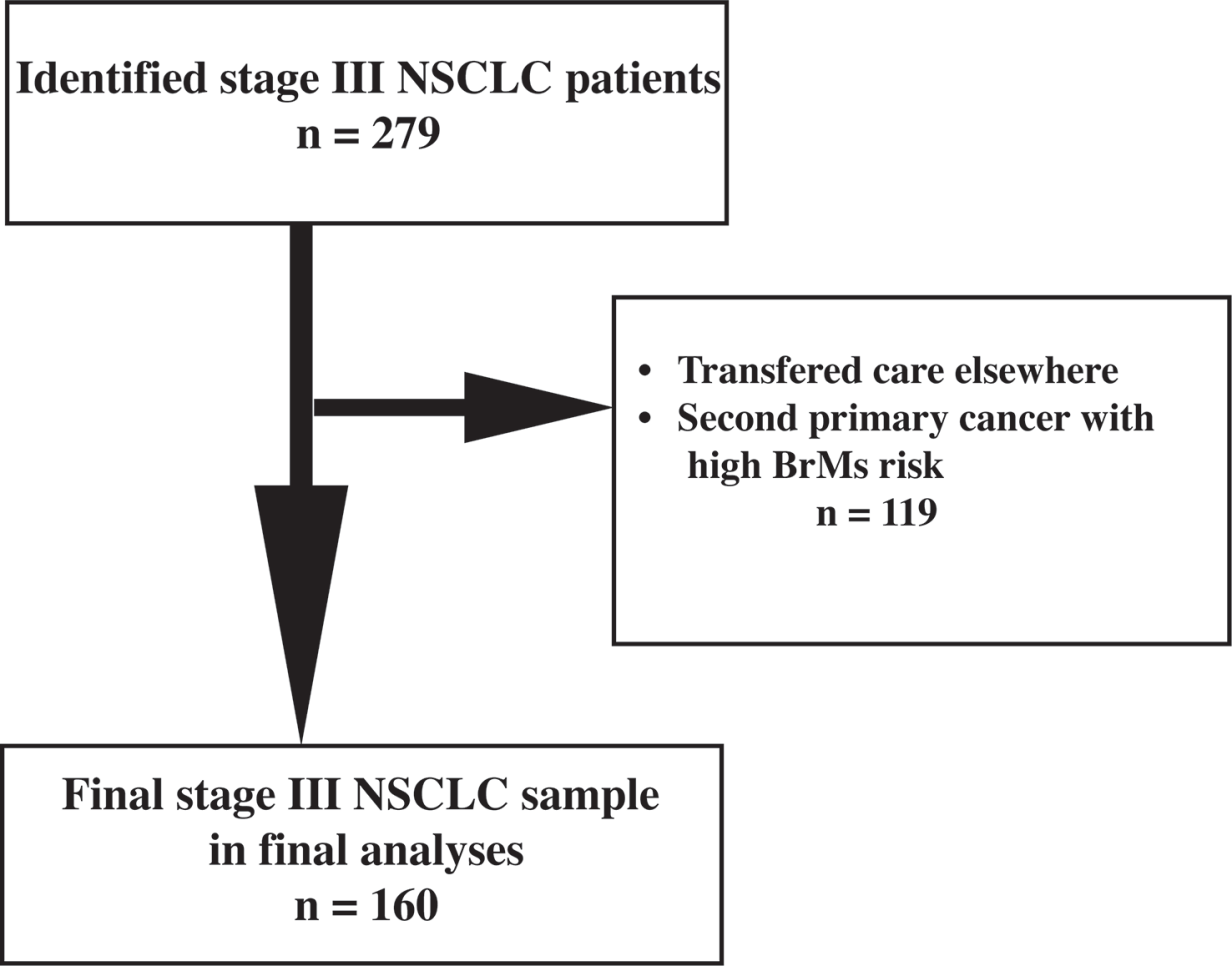
Flowchart indicating the selection process of the final study sample of stage III NSCLC. BrMs: brain metastases.

In this single-center retrospective study, we identified 279 patients who were seen at the Stanford Cancer Center from 2008-2018 and had a confirmed diagnosis of stage IIIA/B/C NSCLC (AJCC 8^th^ edition staging criteria). All patients whose treatment plan included radiation with curative intent were included, regardless of treatment completion. The following information was collected: age at diagnosis of stage III NSCLC, diagnostic imaging findings [including computerized tomography (CT) and whole-body positron emission tomography (PET) scans, and brain MRI], pathology results, disease staging, radiation, and treatment plans [including whether prophylactic cranial irradiation (PCI) was utilized for BrMs prevention], and available follow-up visit information within the prior 6 months, or date of death. A total of 160 stage III NSCLC had complete data and were included in the final analyses of this study. Patients who completed treatment and surveillance elsewhere, did not have available follow-up data, or had a second primary cancer with high potential for brain metastatic disease (e.g., triple negative breast cancer), were excluded (see **Figure 1**).

### Ethics

This retrospective study was approved by Stanford University IRB (reference: IRB-44962).

### Data and statistical analysis

The following time to event outcomes were assessed and analyzed for the entire cohort as well as for each NSCLC histology subtype. The time to overall survival (OS) outcome was summarized using Kaplan-Meier curves. The time to OS was defined as the time from date of diagnosis until death from any cause, and patients who were still alive at the completion of analysis were censored at the date of the last follow up. The time to BrMs outcome was analyzed using competing risk methods and summarized using cumulative incidence curves. Time to BrMs was defined as the time from date of diagnosis until date of BrMs with death as a competing event. Patients who experienced neither BrMs nor death were censored at the date of last follow up. The cumulative incidence of BrMs was calculated over a 2-year period. The OS from date of BrMs was defined as the time from date of BrMs until death from any cause, and patients who were still alive were censored at the date of the last follow up. The “number needed to scan” to detect asymptomatic BrMs was calculated as the total number of patients needed to get surveillance brain MRI to detect asymptomatic BrMs for the entire cohort and for patients with adenocarcinoma subtype (the most common histopathology subtype).

Descriptive statistics were utilized to summarize the characteristics of the study cohort. Chi-square tests were applied to assess outcome differences between histology subtypes. All tests were two-sided with an alpha level of 0.05. All analyses were performed in SAS v9.4 (SAS Institute Inc, Cary, NC).

## Results

A total of 160 patients (93 male and 67 females) with stage III NSCLC were included in the final analyses of this study, see **Figure 1**. Baseline characteristics of the study cohort are described in **Table 1**. The median age at diagnosis of stage III NSCLC was 67 years (range, 34-90 years). Adenocarcinoma was the most common histopathology subtype and was identified in 96 patients (60%), with squamous cell carcinoma in 53 patients (33%). The remaining 11 patients (7%) had other histology subtypes, including large cell and neuro-endocrine tumor. Based on staging work-up, 90 patients (56%) had stage IIIA, 67 patients (42%) had stage IIIB, and 3 patients (2%) had stage IIIC.

**Table 1:** Demographic and baseline characteristics of included stage III NSCLC patients.

	Number (%)
Gender: number (%)	
Male	93 (58%)
Female	67 (42%)
**Age at diagnosis:** median [range] in years	67 [34-90] years
Stage: number (%)	
IIIA	90 (56%)
IIIB	67 (42%)
IIIC	3 (2%)
Histology subtype: number (%)	
Adenocarcinoma	96 (60%)
Squamous cell carcinoma	53 (33%)
Other (including large cell and neuro-endocrine tumor)	11 (7%)
Received PCI: number (%)	
Yes	2 (1%)
No	158 (99%)
Received surveillance brain MRI: number (%)	
Yes	23 (14.4%)
No	137 (85.6%)
Patients who developed BrMs: number (%)	
All histology subtypes	37 of 160 (23%)
Adenocarcinoma	29 of 96 (30%)
Squamous cell carcinoma	6 of 53 (11%)
Other histology types	2 of 11 (18%)
Symptomatic	17 (45.9%)
Asymptomatic	20 (54.1%)
**Number of BrMs**	18 (48.7%)
1	8 (21.6%)
2-3	11 (29.7%)
>3	
OS: median (95% CI) in months	
All histology subtypes	41 (28-53)
Adenocarcinoma	51 (36-68)*
Squamous cell carcinoma	22 (14-43)
Other histology types	21 (10-NR)
Time from BrMs to death: median (95% CI) in months	
All histology subtypes	19 (9-21)
Adenocarcinoma	20 (7-21)
Squamous cell carcinoma	16.5 (1-NR)
Other histology types	64.5 (15-NR)

BrMs, brain metastases; NR, not reached; MRI, magnetic resonance imaging; OS, overall survival; PCI, prophylactic cranial irradiation. *p < 0.01 (adenocarcinoma vs. squamous cell carcinoma subtype).

**Figure 2:**
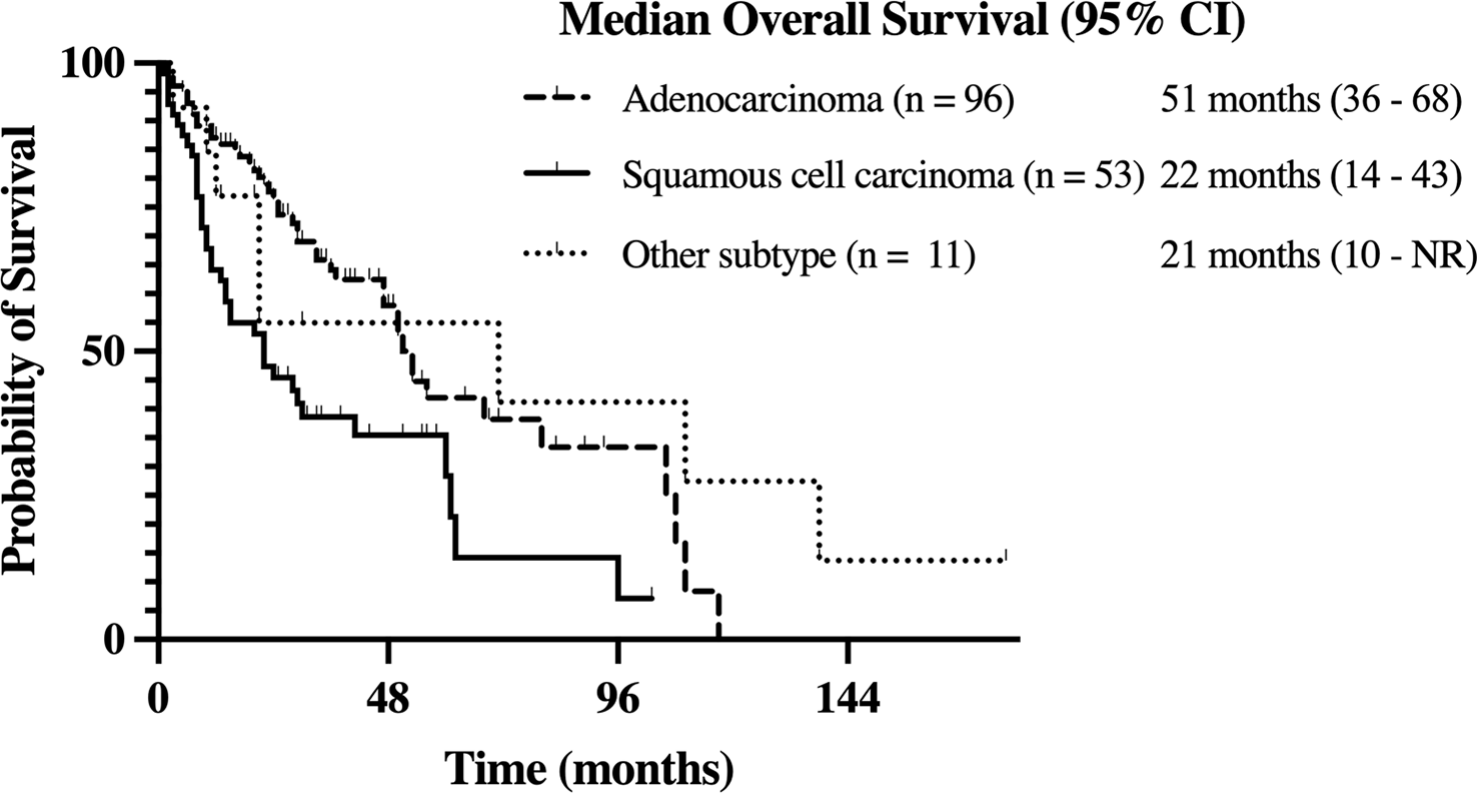
Kaplan-Meier estimate of overall survival (OS) in stage III NSCLC patients with adenocarcinoma, squamous cell carcinoma, and other history types.

The median OS from time of diagnosis for the entire cohort was 41 (95% CI, 28-53) months. In patients with adenocarcinoma, the median OS was 51 (95% CI, 36-68) months. Meanwhile, the median OS was 22 (95% CI, 14-43) months for those with squamous cell carcinoma and 21 (95% CI, 10-NR) months for those with other histology subtypes. The higher OS in patients with adenocarcinoma relative to patients with squamous cell carcinoma was statistically significant (*p* < 0.01; see **Figure 2**).

### Prophylactic cranial irradiation and surveillance for BrMs in stage III NSCLC

Of the entire cohort, only two patients (1%) received PCI at an outside institution prior to establishing care at our cancer center. Neither of these two patients developed BrMs. Institutionally, we offer surveillance brain MRI at 6, 12, and 24 months after completion of treatment. Twenty-three patients (14.4%) received planned surveillance brain MRI to screen for asymptomatic BrMs. For the remaining patients (n = 137, 85.6%), in accordance with current guidelines (8, 9), no planned BrMs surveillance was carried out. Instead, brain MRI was only obtained at systemic recurrence for restaging or on development of neurological symptoms.

### Incidence of BrMs in stage III NSCLC

**Figure 3:**
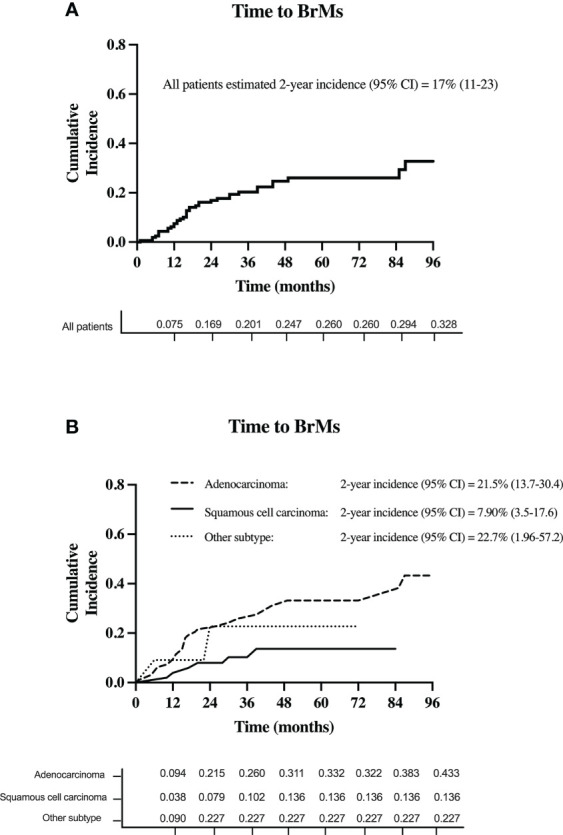
The cumulative incidence of BrMs in **(A)** all patients with stage III NSCLC and **(B)** for each stage III NSCLC histology subtype.

A total of 37 patients (23%) developed BrMs. Of those, 20 (54.1%) were asymptomatic. On initial BrMs identification, 18 patients (48.7%) had one brain metastasis. Meanwhile, 8 patients (21.6%) had 2-3 BrMs and 11 (29.7%) had >3 BrMs. The estimated 2-year cumulative incidence of BrMs was 17% (95% CI, 11-23) for the entire cohort, see **Figure 3A**.

Based on histology subtype, 29 patients with adenocarcinoma (total n = 96; 30.2%) developed BrMs. Of the 53 patients with squamous cell carcinoma, 6 (11.3%) developed BrMs. Meanwhile, in the 11 patients with other histology subtypes, BrMs was identified in 2 (18%) patients. The higher incidence of BrMs in patients with adenocarcinoma [2-year BrMs incidence was 21.5% (13.7-30.4)], compared to squamous cell carcinoma [2-year BrMs incidence was 7.9% (3.5-17.6)], was statistically significant (*p* < 0.01), see **Figure 3B**.

The median time from BrMs development to death was 19 months (95% CI, 9-21) for all patients. No significant differences were noted in time from BrMs to death between NSCLC histology subtypes (see **Table 1**).

### BrMs treatment modality

In patients with BrMs (n = 37), stereotactic radiosurgery (SRS) was utilized to treat 29 patients (78.4%) and whole brain radiation therapy (WBRT) was used to treat 3 patients (8.1%). The remaining 5 patients (13.5%) were treated with systemic therapies alone, without any form of radiation therapy, and were clinically monitored.

### “Number needed to scan” to detect asymptomatic BrMs in stage III NSCLC

Among patients who received no BrMs surveillance (n = 137), 31 developed BrMs (22.6%), 17 of whom were asymptomatic. Meanwhile, among the 23 patients who received surveillance brain MRI, 6 developed BrMs (26.1%), of whom 3 were asymptomatic (see **Table 2**). There was no statistically significant difference in the incidence of BrMs between patients who received BrMs surveillance and those who did not. However, a trend of higher 2-year incidence of BrMs was noted in those received BrMs surveillance [2-year BrMs incidence was 28.5% (11.1-48.9)], compared to patients who did not [2-year BrMs incidence was 15.0% (9.6-21.7)], indicating earlier detection of BrMs (see **Table 2**).

**Table 2:** Comparisons of stage III NSCLC patients who received surveillance brain MRI and those who did not.

	No Surveillance brain MRI (n = 137)	Surveillance brain MRI (n = 23)
Developed BrMs: number (%)		
**Yes**	31 (22.6%)	6 (26.1%)
**No**	106 (77.4%)	17 (73.9%)
**Incidence of BrMs at 2-years:** % (95% CI)	15.0% (9.6-21.7)	28.5% (11.1-48.9)^ns^
BrMs: number (%)		
Symptomatic	14 (45.2%)	3 (50.0%)
Asymptomatic	17 (54.8%)	3 (50.0%)

BrMs, brain metastases; ns, non-significant.

Based on the total number of patients who did not develop BrMs (n = 123), the number of patients needed to receive surveillance brain MRI scans to detect 1 asymptomatic BrMs is 7 for all histology subtypes.

Focusing on adenocarcinoma patients (the most common histology subtype in stage III NSCLC, n = 96), 79 (82.3%) received no BrMs surveillance. Meanwhile, 17 (17.7%) patients received surveillance brain MRI according to our local practice. Among adenocarcinoma patients who received no BrMs surveillance (n = 79), 25 developed BrMs (31.6%), 13 of whom were asymptomatic. In the 17 adenocarcinoma patients who received surveillance brain MRI, 4 developed BrMs (23.5%), of whom 1 was asymptomatic (**Table 3**). Based on the total number of adenocarcinoma patients who did not develop BrMs (n = 67), the number of adenocarcinoma patients needed to receive surveillance brain MRI scans to detect 1 asymptomatic BrMs is 5.

**Table 3:** Comparisons of stage III NSCLC patients with adenocarcinoma subtype who received surveillance brain MRI and those who did not.

	No Surveillance brain MRI (n = 79)	Surveillance brain MRI (n = 17)
Developed BrMs: number (%)		
**Yes**	25 (31.6%)	4 (23.5%)
**No**	54 (68.4%)	13 (76.5%)
BrMs: number (%)		
Symptomatic	12 (48.0%)	3 (75.0%)
Asymptomatic	13 (52.0%)	1 (25.0%)

BrMs, brain metastases.

## Discussion

In this single-center study, we assessed the real-world incidence of BrMs in stage III NSCLC in the modern era of cancer staging and rapid access to advanced diagnostic imaging, including whole body PET/CT scans and brain MRI. The estimated 2-year incidence of BrMs in stage III NSCLC patients treated at our center was 17%. This estimated BrMs incidence is lower than historically described (4). Yet, it is remarkably higher than that reported in more recent clinical trials, including the PACIFIC trial (7). This new evidence of BrMs incidence in stage III NSCLC highlights the considerable difference between outcomes and surveillance practices in the real-world setting versus clinical trials settings.

Due to increasing life expectancy of cancer patients and advances in diagnostic imaging, the incidence of BrMs in all cancer patients (in particular asymptomatic BrMs) is generally increasing (4). Interestingly, based on more recent evidence, the incidence of BrMs in stage III NSCLC has however been declining over time (7). This has been attributed to advances in NSCLC treatments and availability of newer and targeted therapies that has better CNS penetration, such as next-generation tyrosine kinase inhibitors (TKIs), small molecules like pemetrexed, and checkpoint inhibitors (10, 11). More importantly, better access to neuroimaging in recent years has allowed earlier detection of asymptomatic BrMs at initial disease staging, minimizing the possibility of patients being classified as having stage III NSCLC at the time of diagnosis.

Although our sample was likely enriched for targetable mutations (such as EGFR and ALK rearrangements), it remains a close representation of the real-world stage III NSCLC patient population treated at specialized cancer centers, with adenocarcinoma and squamous cell carcinoma histology subtypes comprising 60% and 33% of our cohort respectively. The incidence of BrMs was higher in patients with adenocarcinoma relative to other histology subtypes, with 30% of our patients with adenocarcinoma subtype developing BrMs. This is in alignment with the evidence of higher BrMs rates in adenocarcinoma subtype, especially in those harboring targetable mutations, such as EGFR and ALK rearrangements (12–16). Likewise, the significantly higher OS we observed in our patients with adenocarcinoma compared to squamous cell carcinoma histology subtype is consistent with recent literature (12, 17).

The clinical practice of BrMs surveillance in stage III NSCLC remains controversial. Surveillance brain MRIs are not explicitly recommended and current stage III NSCLC guidelines, including the 2020 National Comprehensive Cancer Network (NCCN) guidelines (9), recommend obtaining brain MRI to detect asymptomatic BrMs only at the time of initial diagnosis or systemic recurrence. It is unclear however how closely physicians adhere to these guidelines in the real-world setting (18). According to our local practice, 14.4% of (n = 23) of our stage III NSCLC patients received planned surveillance brain MRIs to screen for asymptomatic BrMs at 6, 12, and 24 months after completion of treatment. Most patients (85.6%) however were monitored clinically and only received surveillance brain MRI at the time of systemic recurrence as part of their re-staging process. The frequency of asymptomatic BrMs among patients who received BrMs surveillance was 13%, which was nearly equivalent to the frequency of asymptomatic BrMs among patients who did not receive surveillance brain MRI (12.4%). Although this is not surprising, a trend of earlier BrMs detection was noted in those who received BrMs surveillance. Interestingly, 50% of the patients who developed BrMs while undergoing BrMs surveillance required an additional brain MRI due to developing new neurological symptoms, indicating that regular surveillance was not entirely successful in detecting asymptomatic BrMs early. Overall, we estimated that 7 patients with stage III NSCLC “needed to be scanned” to detect one asymptomatic BrMs. Based on these observation, routine BrMs surveillance is likely to be challenged by its lack of superiority to clinical monitoring and potential cost-ineffectiveness, especially in less resourced areas and low-income countries. However, with ready access to, and growing affordability of, brain MRI, it can be argued that early BrMs detection is very pertinent to treatment planning and delaying BrMs-related complications. The benefit of BrMs surveillance might thus be justifiable in selected stage III NSCLC patients, such as patients with adenocarcinoma, those at high-risk of recurrence (including those harboring targetable mutations such as EGFR and ALK), and those with programmed cell death ligand 1 (PD-L1) expression on less than 1% of tumor cells (19, 20).

Recently, our group reviewed the landmark trials that investigated PCI in stage III NSCLC (10). Despite the fact that all, but one (21), of these trials were able to demonstrate a significant reduction in the incidence of BrMs in stage III NSCLC with PCI (22–28), they consistently demonstrated lack of significant benefit on OS. In the 2019 update to the Radiation Therapy Oncology Group (RTOG) 0214 phase 3 randomized clinical trial (RCT), the largest RTC of PCI in stage III NSCLC, the 5-year incidence of BrMs was 28.3% in the observation group compared to 16.7% in the PCI group (HR 0.43, p=0.003) (27). Despite this seemingly promising result, the findings unfortunately did not translate to an improvement in OS (neither 5-year, 10-year, nor median OS was significantly different between the two groups) (27). Interestingly, compared to our study, the 2-year incidence of BrMs in the NRG Oncology-RTOG 0214 Phase 3 RCT was 24.3% in the observation group (relative to 10.9% in the PCI group) (27). The higher 2-year BrMs incidence in the trial’s observation group likely reflects the study accrual period between 2002 and 2007 and does not appear entirely consistent with contemporary evidence of lower BrMs incidence in stage III NSCLC or the findings of the current study (7). In our cohort, only 2 patients (1%) received PCI at other institutions prior to establishing care at our center. Neurological complications associated with WBRT are well documented and range from long-term cognitive dysfunction, gait and motor disturbance, and urinary incontinence (29, 30). These detrimental neurological complications and the lack of significant benefit on survival outcomes argue against PCI application in stage III NSCLC.

This study has several limitations. Firstly, this is a retrospective single center study and thus the results are subject to bias. Compared to clinical trials settings, however, we believe this retrospective study was less limited by the strict inclusion/exclusion criteria often applied in clinical trials and thus had more potential to estimate a closer real-world incidence of BrMs. For example, patients participating in the PACIFIC trial successfully completed chemoradiation without disease progression prior to enrollment, whereas patients in this cohort were included if their treatment plan had curative intention, regardless of completion. Secondly, molecular testing (such as EGFR/ALK mutations status) was not available for all patients in our cohort and thus this relevant information was not incorporated in our analyses. Future studies are strongly encouraged to include the results of targetable mutations testing when assessing the association between stage III NSCLC BrMs predisposition and clinical outcomes. Thirdly, we considered the fact that the American Joint Committee on Cancer (AJCC) Classification of NSCLC underwent edition changes during our study period which may affect classification of stage III NSCLC patients. Although, we do not believe this had largely impacted our data as most of the study period comprised the 7^th^ edition classification system. Lastly, given that our center is a major academic center, there is a possibility that most of the patients who continued long-term follow-up represent a population of patients that are often doing clinically better than those who were lost to follow-up.

## Conclusion

In this retrospective single-center study, the 2-year incidence of BrMs in stage III NSCLC was 17%. This BrMs incidence is lower than historically reported, but higher than that reported in recent clinical trials. Although, we found surveillance brain MRI not superior to clinical monitoring in detecting asymptomatic BrMs, a trend of earlier BrMs detection was noted. Therefore, surveillance brain MRI may still be appropriate in selected subgroups of stage III NSCLC patients, such as patients with adenocarcinoma and those with high risk of recurrence. At our institution, PCI is not performed for stage III NSCLC and there is a clear preference to deliver SRS over WBRT to treat BrMs.

## Data availability statement

The raw data supporting the conclusions of this article will be made available by the authors, without undue reservation.

## Author contributions

SA, TL, RK, KT, BL, JN, SN: Conceptualization, methodology, writing, investigation, editing. RE, MD, SS, EP, AC, LV, HW, SP, KR: Writing, visualization, diting. All authors contributed to the article and approved the submitted version.
